# Sinus pause during nerve integrity monitoring tube insertion following anesthetic induction with a low-dose neuromuscular blocking agent: A CARE-compliant case report

**DOI:** 10.1097/MD.0000000000026683

**Published:** 2021-07-23

**Authors:** Sung-Hye Byun, Jae-Min Jeon

**Affiliations:** Department of Anesthesiology and Pain Medicine, School of Medicine, Kyungpook National University, Daegu, Republic of Korea.

**Keywords:** intraoperative nerve monitoring, intubation, neuromuscular blocking agents, onset time, sinus pause

## Abstract

**Rationale:**

Nerve integrity monitoring (NIM) tubes are commonly used in thyroid surgery to prevent recurrent laryngeal nerve injury. To achieve the optimal electromyographic signal for NIM as intraoperative neural monitoring (IONM), the neuromuscular blocking agent (NMBA) dose should be low. The use of a low-dose NMBA increases the anesthetic and analgesic agent dose required to attenuate the laryngeal reflex during intubation. In addition, since the NMBA onset time is delayed, depending on the situation, anesthesia may become excessively deep or shallow before intubation.

**Patient's concern:**

A 51-year-old woman scheduled for thyroid lobectomy received 0.3 mg/kg of rocuronium. Three minutes later, when the NIM tube was inserted through the vocal cord, the patient's heart rate (HR) was undetectable for 2 seconds.

**Diagnosis:**

We suspected that the use of a high-dose anesthetic agent and remifentanil or the laryngocardiac reflex induced the sinus pause.

**Interventions:**

To maintain the anesthetic depth, we administered 6 vol% of desflurane. Because the patient's systolic blood pressure was 70 mmHg and HR was 30 beats/min, we discontinued the remifentanil infusion and administered 8 mg of ephedrine.

**Outcomes:**

The patient's vital signs recovered to normal levels. Subsequently, there were no episodes of bradycardia or arrhythmia.

**Conclusion:**

Sinus pause or severe bradycardia may occur due to the laryngocardiac reflex or the administration of a high-dose anesthetic and analgesic agent during tracheal intubation in patients who received a low-dose NMBA for IONM induction using an NIM tube. Anesthesiologists should be aware of these risks and take precautions to maintain adequate anesthesia, be prepared to administer vasoactive drugs to increase the blood pressure and HR if needed, and, if possible, intravenously administer lidocaine to attenuate the laryngeal reflex during intubation.

## 1 Introduction

Nerve integrity monitoring (NIM) tubes are increasingly being used to prevent recurrent laryngeal nerve (RLN) injury during thyroid surgery. Although there is insufficient evidence supporting the routine use of NIM tubes and its potential benefit remains debatable,^[1]^ many surgeons still consider it to be important because it enables quick and reliable nerve identification and postoperative verification of RLN function.^[2]^ NIM tubes are used to observe electromyographic (EMG) responses to direct electrical RLN stimulation. Therefore, using a neuromuscular blocking agent (NMBA) may lower its sensitivity to nerve injury^[2]^ by affecting the EMG signals and intraoperative neural monitoring (IONM) results and interpretation.^[3]^

Herein, we report a case of sinus pause following the use of a low-dose NMBA during NIM tube insertion for IONM. We discuss the possible causes and suggest precautions that should be taken during NIM tube insertion to prevent such a situation.

## 2 Case presentation

A 51-year-old woman (height: 155.4 cm, actual body weight: 59.4 kg, ideal body weight: 48 kg) was diagnosed with thyroid papillary cancer and scheduled for thyroid lobectomy. She did not have any preexisting conditions or factors that could cause a difficult airway, and her dental condition was good. No notable findings were observed on preoperative evaluation. The thyroid surgeon decided to use an NIM system (NIM-Neuro 3.0 System, Medtronic Xomed Inc., Jacksonville, FL) for RLN monitoring and asked the anesthesiologist to use an NIM tube (NIM EMG Endotracheal Tube, Medtronic Xomed Inc., Jacksonville, FL) using a small dose of the NMBA.

The patient was not premedicated. On the patient's arrival at the operating room, standard monitoring was initiated, which included non-invasive blood pressure (BP), electrocardiogram, and peripheral oxygen saturation monitoring. The depth of anesthesia was monitored using the bispectral index, with the sensor placed on the forehead. The patient was preoxygenated with 100% oxygen using a face mask, and we intravenously infused 40 mg of lidocaine and remifentanil with a target effect-site concentration of 2.0 ng/ml. Since the patient's ideal body weight was 48 kg, we administered 100 mg of propofol. After the bispectral index dropped to below 60, we administered 0.3 mg/kg (ED95) of rocuronium, as per our center's protocol. We performed bag-and-mask ventilation for 3 minutes in anticipation of the onset of rocuronium action, which was delayed. Anesthesia was maintained with 7 vol% of desflurane, and the target concentration of remifentanil was increased to 4.0 ng/ml. The patient's systolic blood pressure (SBP) was 100 mmHg and heart rate (HR) gradually decreased from 70 to 50 beats/min.

Three minutes after rocuronium administration, we performed tracheal intubation using a Macintosh laryngoscope. We indirectly lifted the epiglottis by placing the blade tip of the laryngoscope on the vallecula and obtained a Cormack–Lehane grade 1 laryngeal view. An NIM tube (internal diameter, 6.0 mm; outer diameter, 8.8 mm) was slightly bent at the tip by inserting a stylet and was placed over the glottis under laryngoscopic guidance. After removing the stylet, the NIM tube was inserted beyond the vocal cord. At that moment, the patient's heart stopped beating for 2 seconds. The NIM tube was not removed as the patient had been successfully intubated. To maintain the depth of anesthesia, 6 vol% of desflurane was administered, and the remifentanil infusion was discontinued. The patient's SBP was 70 mmHg and HR was 30 beats/min. Hence, 8 mg of ephedrine was intravenously injected, following which the patient's vital signs recovered to normal levels. Bradycardia or arrhythmia did not subsequently occur, and the vital signs were stable; thus, we proceeded with the surgery, which was completed without complications. Ethical approval was not required by the institutional review board as the clinical data were deidentified; however, the patient has provided informed consent for the publication of this case report.

## 3 Discussion

The fundamental problem in this case was the use of a small dose of the NMBA. Consequently, additional intervention was required to attenuate the response to stimuli evoked by laryngoscopy and intubation, and the onset of NMBA action was delayed, leading to a delay in achieving the optimal condition for performing intubation. To attenuate the response to stimuli on the airway during intubation, we administered 6 to 7 vol% of desflurane in addition to the induction dose of propofol, and the target effect-site concentration of remifentanil was increased to 4 ng/ml. A greater opioid or propofol dose is required for satisfactory intubation when NMBA is not used during anesthesia induction than when NMBA is used,^[4]^ and this may lead to a drop in the BP and HR. Intubation can be performed within 60 to 90 seconds of administration of twice the ED95 (0.6 mg/kg) of rocuronium. However, it takes 90 to 180 seconds to reach the optimal intubation condition when a lower dose (0.45 mg/kg) is used due to delayed onset of NMBA action.^[5–7]^ It has been reported to take 4 minutes to reach a satisfactory intubation condition when the dose is reduced to the ED95 (0.3 mg/kg).^[8]^ Further, the onset of rocuronium action may be further delayed if the cardiac output is reduced due to administration of high doses of anesthetic and analgesic agents.^[9]^

Delayed onset of NMBA action may lead to one of two contrary scenarios. In the first scenario, a higher dose of propofol or inhaled agent and opioid may be infused for a longer duration than is conventionally performed during intubation without stimulation. Our patient received these agents for a relatively long duration (3 minutes), and this may have caused the drop in their BP and HR. Sato and Higuchi et al. reported cases of sinus arrest and asystole following anesthesia induction with high-dose remifentanil in patients with sick sinus syndrome and in patients who were receiving beta-adrenergic, calcium channel, and angiotensin II receptor blocking agents.^[10,11]^ Remifentanil has been known to reduce HR by directly inhibiting sinus node function and causing vagal stimulation.^[12]^ Our patient did not have any known underlying disease and was not on any medication. However, bolus doses of remifentanil can induce severe bradycardia even in healthy people, and greater precautions are needed for patients with sinus node dysfunction, as these patients are highly susceptible to remifentanil-induced suppression. In the second scenario, the superior laryngeal nerve may be stimulated during laryngoscopy or tube insertion during intubation if anesthesia has not been adequately maintained while waiting for the onset of NMBA action. When anesthesia is maintained using inhaled anesthetic agents as in our patient's case, if adequate ventilation is not maintained, the depth of anesthesia may be reduced, and this may induce the laryngocardiac reflex.^[13]^ The laryngocardiac reflex is a vagal reflex induced by the activation of afferent parasympathetic nerve fibers following stimulation of the lower pharynx, larynx, trachea, and epiglottis. It may be triggered when the laryngeal surface of the epiglottis or supraglottic area is lifted using the blade tip of a suspension laryngoscope. The sensory distribution of the vagus nerve is more focused on the supraglottic area and laryngeal surface of the epiglottis compared to the base of the tongue, the pharyngeal surface of epiglottis, which is where the curved blade of the laryngoscope is placed during intubation. Although our patient underwent usual process of intubation using curved blade Macintosh laryngoscope, the use of a slightly larger NIM tube might have led to the stimulation of the structures around the epiglottis and induced the laryngocardiac reflex. As in our patient's case, the vital signs may immediately recover without any problems; however, there are reports of cases in which cardiopulmonary resuscitation was needed.^[14,15]^ Therefore, anesthesiologists should be aware of the possibility of induction of the laryngocardiac reflex during intubation.

One method to prevent these scenarios is to use a sufficient dose of the NMBA. To ensure that the patient is fully relaxed, many centers administer the conventional NMBA dose (0.6–0.9 mg/kg), which is 2 or 3 times the ED95, for tracheal intubation during anesthesia induction, and it is discontinued after successful intubation.^[2,16]^ The use of a sufficient dose of the NMBA leads to a good intubation condition and reduced post-intubation laryngotracheal symptoms. It also decreases the amount of anesthetic and analgesic required and thus is conducive to hemodynamic stability.^[17]^ As regimens of NMBA during IONM have been extensively documented in the literature,^[18]^ we will not describe them in this report. At our hospital, anesthesiologists use a small dose of the NMBA during anesthesia induction to confirm that the NIM tube has been placed appropriately before beginning the surgery. If a low dose of the NMBA must be used, as is the protocol of our hospital, topical anesthesia can be considered for attenuating airway stimulation and preventing the abovementioned risks. The use of 4% laryngotracheal lidocaine has been reported to lead to an improved intubation condition and a stable hemodynamic condition.^[19]^ However, studies mention that the use of lubricants such as topical lidocaine should be avoided when using an NIM tube, although this does not alter the EMG signals.^[2,16]^ It has also been reported that intravenous administration of 2 mg/kg of lidocaine significantly attenuated the laryngeal reflex in anesthetized children, although this effect was short-lived.^[20]^ Furthermore, a report exists of a patient in whom a NMBA was not used for IONM; in that patient, intravenous infusion of 1.5 mg/kg/h of lidocaine following a bolus dose of 1 mg/kg led to a gradual attenuation of the laryngotracheal reflex, and the surgery that was frequently held up by coughing could be resumed.^[21]^ In addition, anesthesia must be adequately maintained to prevent inducing the airway reflex. Therefore, if there is a delay between the administration of the induction dose and intubation, an additional dose of the anesthetic agent should be continuously infused or total intravenous anesthesia should be performed from the beginning of the surgery. If an inhaled agent is used, as was the case here, the end-tidal anesthetic concentration must be monitored. The use of total intravenous anesthesia for IONM is recommended because halogenated inhaled agents reduce EMG amplitude and increase latency in a dose-dependent manner, and nitrous oxide acts synergistically to inhaled agents and most intravenous anesthetics.^[16]^ However, the guideline statement has also reported that inhaled agents may be used.^[2]^ Finally, when using relatively high doses of anesthetics and analgesics, the patient's BP and HR must be carefully monitored during induction, and sympathomimetic drugs such as ephedrine or anti-cholinergic drugs should be readily available for immediate use upon need.

In conclusion, sinus pause or severe bradycardia may occur due to several reasons during laryngoscopy and tracheal intubation in patients receiving low-dose NMBAs for IONM using an NIM tube. It is important to be aware of these risks and consider strategies to prevent them. Anesthesiologists should take precautions to maintain adequate anesthesia, be prepared to administer vasoactive drugs to increase the BP and HR if needed, and, if possible, intravenously administer lidocaine to attenuate the laryngeal reflex during intubation.

## Author contributions

**Conceptualization:** Sung-Hye Byun.

**Data curation:** Sung-Hye Byun.

**Investigation:** Jae-Min Jeon.

**Resources:** Jae-Min Jeon.

**Supervision:** Sung-Hye Byun.

**Writing – original draft:** Sung-Hye Byun.

**Writing – review & editing:** Sung-Hye Byun, Jae-Min Jeon.
